# Prescribing vs. Dispensing

**Published:** 1907-10

**Authors:** 


### Prescribing vs. Dispensing.

The doctor who enters the chamber of suffering at the hour of midnight, armed only with a pencil and a piece of paper, is as unwise as the soldier who rushes to repel the midnight attack of the enemy leaving his arms and ammunition in his tent. A drug administered at once by the hand of the physician to a patient in dire distress is certainly more satisfactory to both the physician and patient than a prescription written on a piece of paper. In a few minutes both the physician and the prescription are gone and the time of their return in doubt.

The general practitioner should keep on hand remedies for the relief of severe pain, emptying the stomach and bowels quickly, antidotes to carbolic acid and a few of the more popular poisons. Heart stimulants, aromatic spirit of ammonia, strychnine, nitroglycerine, amyl nitrite, alcohol if to hand, are at times and under circumstances, worth their weight in gold. The general practitioner should neither dispense nor prescribe exclusively, but adhere to the happy medium doing part of each, as best for both patient and himself.

Dispensing physicians have more than once been the object of legislative attack, but the universal decision has always been that the right to furnish medicines to his patients is inherent in the physician, and it is not probable that the legislature will attempt to deprive him of it or that the court would sustain any such an attempt if made.

The writer while not anxious for or expecting the job if he were ever called upon to get up a revised edition of the human frame, would put the calf of the leg in front of the shin bone, enlarge the rectum, excise the bladder and turn the ureters into the rectum, thus giving a person two or three loose stools per day, chicken like, do away with constipation and cease making a sewer of the sexual organs. The druggist had some very fine painkiller, his own make, which he recommended very highly to his undertaker neighbor saying: "If you take this once you will never take any other." "No, thank you," said the undertaker, "I do not care for any, but I will recommend it to all my acquaintances."

